# DFS70/LEDGFp75: An Enigmatic Autoantigen at the Interface between Autoimmunity, AIDS, and Cancer

**DOI:** 10.3389/fimmu.2015.00116

**Published:** 2015-03-20

**Authors:** Anamika Basu, Tino W. Sanchez, Carlos A. Casiano

**Affiliations:** ^1^Center for Health Disparities and Molecular Medicine, Department of Basic Sciences, Loma Linda University School of Medicine, Loma Linda, CA, USA; ^2^Department of Medicine, Loma Linda University School of Medicine, Loma Linda, CA, USA

**Keywords:** autoantibodies, LEDGFp75, autoantigens, HIV, cancer, autoimmunity, DFS70

## Abstract

Clinical and diagnostic laboratories often encounter patient sera containing antinuclear antibodies (ANAs) that produce a nuclear dense fine speckled immunofluorescence pattern on HEp-2 cells. These autoantibodies usually target the dense fine speckled protein of 70 kDa (DFS70), commonly known as lens epithelium-derived growth factor p75 (LEDGFp75). Anti-DFS70/LEDGFp75 autoantibodies have recently attracted much interest because of their relatively common occurrence in sera from patients with positive ANA tests with no clinical evidence of systemic autoimmune rheumatic disease (SARD). Their presence has been documented primarily in patients with diverse non-SARD inflammatory conditions and “apparently healthy” individuals. While there is circumstantial evidence that depending on the context these autoantibodies could play protective, pathogenic, or sensor roles, their significance remains elusive. DFS70/LEDGFp75 has emerged during the past decade as a stress transcription co-activator relevant to HIV integration, cancer, and inflammation. It is not clear, however, what makes this protein the target of such a common autoantibody response. We suggest that a better understanding of DFS70/LEDGFp75 biology is key to elucidating the significance of its associated autoantibodies. Here, we discuss briefly our current understanding of this enigmatic autoantigen and potential scenarios leading to its targeting by the immune system.

## Introduction

The clinical and biological significance of anti-DFS70/LEDGFp75 autoantibodies has been puzzling because in spite of being relatively common and capable of reaching high titers, they lack disease specificity and can be found in “apparently healthy” individuals and in patients with diverse non-SARD inflammatory conditions (1–7). These autoantibodies are present at very low frequencies in patients with systemic autoimmune rheumatic disease (SARD), and their prevalence appears to be higher in “apparently healthy” individuals than in these patients (1, 4–7). When present in SARD patients, these autoantibodies are usually accompanied by other SARD-specific autoantibodies; however, when present as the sole antinuclear antibody (ANA) specificity they tend to exclude a SARD diagnosis (5–7). This led to the hypothesis that anti-DFS70/LEDGFp75 autoantibodies could be useful biomarkers to exclude a SARD diagnosis, and an algorithm for their clinical utility has been developed (3, 6, 7). These unique features have recently attracted much attention, and vigorous efforts to uncover the significance of this autoantigen–autoantibody system are currently underway in several laboratories and clinics around the world.

**Figure 1:**
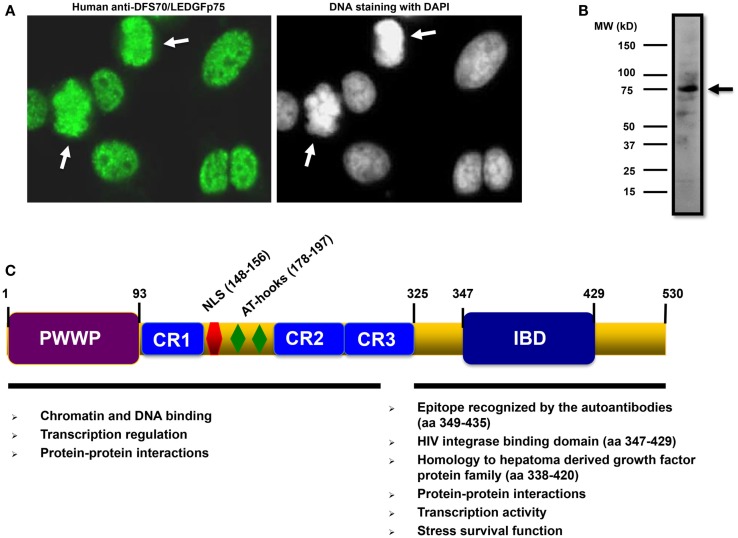
**Features of DFS70/LEDGFp75 and its autoantibodies**. **(A)** Staining pattern of human autoantibodies to DFS70/LEDGFp75 in HEp-2 slides visualized by indirect immunofluorescence (IIF) microscopy. Arrows point to bright staining in condensed metaphase chromosomes. **(B)** Immunoblot showing reactivity of a human serum containing anti-DFS70/LEDGFp75 autoantibodies against total protein lysates from PC3 prostate cancer cells. A prominent band of approximately 75 kDa was observed with the serum. Immunoreactivity was detected by enhanced chemiluminescence. **(C)** Diagram depicting the main structural domains and motifs of DFS70/LEDGFp75 with their proposed functions.

Anti-DFS70/LEDGFp75 autoantibodies are usually detected in standard HEp-2 substrates by indirect immunofluorescence (IIF) microscopy. They are characterized by staining of dense fine speckles (DFS) in the nucleoplasm of cells in interphase, typically excluding the nucleolus, with bright staining of metaphase chromosomes in mitotic cells (Figure 1A). Confirmation of the specificity of the DFS-IIF pattern for DFS70/LEDGFp75 can be achieved by examining the sera for reactivity with a protein band of approximately 70–75 kDa in immunoblots of cell lysates that express high levels of this protein such as cancer cells (8, 9) (Figure 1B). Other detection platforms include ELISA and chemiluminescence immunoassays such as the Quanta Flash-DFS70 (INOVA Diagnostics).

Distinguishing anti-DFS70/LEDGFp75 autoantibodies from other common ANAs could be challenging because if examined by non-expert interpreters in HEp-2 substrates, they could be confused with other nuclear IIF patterns (6). This, combined with the use of single antibody detection assays, has led to inter-laboratory variability in reports of the frequencies of anti-DFS70/LEDGFp75 autoantibodies in different populations and patient cohorts. Confusing the DFS-IIF pattern with other ANA patterns may lead to inaccurate immunodiagnosis of SARD, with ensuing negative consequences for the antibody-positive individuals. Therefore, there is growing consensus that the ANA test should not be used as the sole method for the detection of anti-DFS70/LEDGFp75 autoantibodies, and that confirmatory tests are needed until a highly accurate immunoassay for the detection of these antibodies is developed and well-validated in multiple laboratories.

Our functional and structural understanding of DFS70/LEDG Fp75, also known as transcription co-activator p75 and PC4 and SFRS1 interacting protein 1 (PSIP1), has dramatically increased since its discovery in the late 1990s. This has led to its emergence as a stress response transcription co-activator of growing relevance to human disease, including acquired immunodeficiency syndrome (AIDS), cancer, and diverse inflammatory conditions (8–11). However, in spite of the growing knowledge on DFS70/LEDGFp75 biology, we are still perplexed by the relatively common autoantibody response to this protein among ANA-positive patients who have no clinical evidence of SARD. As briefly discussed below, we believe that integrating our growing knowledge of this DFS70/LEDGFp75 with the emerging observations on the clinical contexts in which its associated autoantibodies arise will help uncover the significance of this autoantigen–autoantibody system.

## DFS70/LEDGFp75 Structure, Expression, and Functions

DFS70/LEDGFp75 is a multi-functional protein with well-defined domains (Figure 1C). Its N-terminal portion encompasses chromatin-binding elements such as a proline–tryptophan–tryptophan–proline (PWWP) domain, charged regions (CR), AT-hook motifs, and a nuclear localization signal (NLS). All these sequences cooperate to facilitate DFS70/LEDGFp75 binding to active transcription sites in the chromatin, where it interacts with RNA polymerase-II transcription complexes to regulate stress gene expression. The C-terminal portion encompasses a highly conserved, conformational, and functional domain that contains the epitope targeted by the autoantibodies (residues 349–435) (Figure 1C) (12). To date, the functions associated with the C-terminal portion of DFS70/LEDGFp75 include transcriptional activity, stress survival activity, and serving as a hub for specific protein–protein interactions (Figure 1C). The immune targeting of this functionally important and highly conserved C-terminal portion of DFS70/LEDGFp75 is consistent with the generalized notion that ANA-defined epitopes typically comprise highly conserved, conformation dependent, functioning sites.

DFS70/LEDGFp75 has a short spliced variant designated LEDGFp52 that corresponds to its N-terminal residues 1–325 plus eight, intron-derived, unique residues 326–333 [reviewed in Ref. (10)]. LEDGFp52 is typically not targeted by the autoantibodies and plays roles in transcription and mRNA splicing. We observed that when overexpressed in cancer cells LEDGFp52 induces apoptosis and antagonizes the transcriptional activity of DFS70/LEDGFp75 [reviewed in Ref. (10)].

Although originally presumed to be a lens epithelial cell (LEC) growth factor, compelling evidence indicates that this autoantigen may not be a growth factor but rather a stress response protein that is ubiquitously expressed in mammalian cells and tissues, with overexpression in cancer cells and tumors (8–10, 13). The Human Protein Atlas lists the *PSIP1/LEDGF* gene as moderately or highly expressed, both at the transcript and protein levels, in 80 of 81 non-cancerous tissues analyzed (http://www.proteinatlas.org). This tissue expression analysis, however, does not distinguish between the p75 and p52 splice variants.

DFS70/LEDGFp75 plays a key role in promoting cell survival in the face of environmental stressors such as alcohol, UVB irradiation, serum starvation, and certain viruses and cytotoxic drugs (9, 10, 13, 14). Ultimately, these stressors lead to increased cellular oxidative stress, resulting in DFS70/LEDGFp75 activation. The stress survival functions of DFS70/LEDGFp75 are linked to its ability to transcriptionally activate stress protective, antioxidant, and inflammatory genes (10, 13, 14). DFS70/LEDGFp75 contributes to the activation of these genes by forming complexes with multiple chromatin-associated proteins. Both the N- and C-terminal portions of DFS70/LEDGFp75 participate in its transcription and stress survival functions. The ability of DFS70/LEDGFp75 to preserve the structural integrity of vital cellular organelles such as the lysosome points to a critical role for this protein in regulating cellular life and death decisions in response to stress [reviewed in Ref. (10)]. For additional details on the main cellular functions ascribed to DFS70/LEDGFp75 please refer to Table 1.

**Table 1:** **Key cellular functions of DFS70/LEDGF/p75**.

Function	Mechanism
Apoptosis signaling	Cleaved during apoptosis by caspases into fragments that retain autoepitope and that persist as cells transition from apoptosis to secondary necrosis; this cleavage abolishes its pro-survival activities
Cellular stress survival	Protects mammalian cells against a variety of environmental stressors (e.g., UVB, oxidative stress, thermal stress, alcohol, certain drugs)
Chemoresistance	Upregulated in chemoresistant cancer cells; promotes lysosomal stability in the context of drug-induced caspase-independent cell death
Chromatin binding	Facilitated by its PWWP domain, AT hooks, and charged regions
Development	Knockout associated with skeletal and craniofacial abnormalities possibly due to deficient activation of *HOX* genes
DNA repair	Component of homologous recombination repair complex
HIV-1 integration	Tethers HIV-integrase to transcriptionally active sites to facilitate integration of HIV-1
Inflammation	Implicated in activation of IL6/STAT3 pathway
Leukemogenesis	Upregulated in chemoresistant leukemia blasts; binds to menin-MLL transcription complex to activate leukemia-associated genes; forms fusion proteins with NUP98 in some leukemia patients
Malignant transformation	Overexpressed in cancer cells and certain solid tumors; promotes cell survival signaling, proliferation, migration, clonogenicity, angiogenesis, and tumor growth
Nuclear import	Mediated by single classical NLS
Protein–protein interactions	Binds to several chromatin-associated proteins (e.g., HIV-IN, MeCP2, MLL-Menin, JPO2) through its PWWP and IBD domains
Transcription	Interacts with the RNA polymerase-II transcription complex; contributes to the activation of stress survival, cancer-associated, inflammation, and *HOX* genes; may also act as repressor depending on context

Knockout of the *PSIP1/LEDGF* gene (which encodes DFS70/LEDGFp75 and p52) in mice, while not embryonically lethal, was associated with craniofacial and skeletal abnormalities that led to premature death in most newborns shortly after birth due to their inability to nurse, possibly because of olfactory dysfunction or motor abnormalities (15). *PSIP/LEDGF*^−/−^ knockout mice that survived to adulthood displayed motor and behavioral defects, craniofacial abnormalities, and eyelid inflammation. These abnormalities were associated with dysregulation of *HOX* genes, many of which were significantly upregulated by the loss of the *PS1P1/LEDGF* gene (15). Interestingly, the *PS1P1/LEDGF* gene has been mapped to chromosome 9p22.2 region, which is adjacent to a locus associated with the 9p deletion syndrome, a rare human chromosomal abnormality characterized by atypical craniofacial features, inability to nurse and breath, eye diseases, and several other anomalies. It remains to be determined if loss of DFS70/LEDGFp75 is a common genetic abnormality in this syndrome.

## DFS70/LEDGFp75 and HIV/AIDS

DFS70/LEDGFp75 is essential for integration of the human immunodeficiency virus 1 (HIV-1), a role that is mediated by its high-affinity interaction with HIV-1 integrase (HIV-IN) (11). HIV-IN binds to a highly conserved, C-terminal domain of DFS70/LEDGFp75 mapped to residues 347–429 and named integrase binding domain (IBD) (11). This interaction stabilizes HIV-IN and contributes to DFS70/LEDGFp75-mediated shuttling of HIV-1 into the nucleus and tethering it to chromatin to promote viral integration to transcriptionally active sites. This key role in HIV-1 integration has catapulted DFS70/LEDGFp75 into the limelight of promising candidates for therapeutic targeting in HIV/AIDS (11).

Remarkably, the DFS70/LEDGFp75 autoepitope region (residues 349–435) is essentially the same region comprised by the IBD (residues 347–429). While the biological significance of these “coincidental” findings is unclear, they raise intriguing questions. Why would an epitope region targeted by autoantibodies be the same region specifically recognized by the HIV-IN? What elements within this region make it attractive for targeting by both the immune system and HIV-1? Would the presence of anti-DFS70/LEDGFp75 autoantibodies, which may absorb extracellularly released DFS70/LEDGFp75, protect individuals against HIV infection by preventing this protein from binding to HIV-IN? Studies with cohorts of HIV-positive patients that are susceptible or resistant to develop full-blown AIDS may provide interesting insights into the possible relationship between the presence of these autoantibodies and disease progression.

## DFS70/LEDGFp75 and Cancer

We reported a significantly higher frequency of autoantibodies to DFS70/LEDGFp75 in prostate cancer patients (PCa) compared to age- and gender-matched controls [reviewed in Ref. (8, 10)]. While several groups have recently confirmed this observation in different PCa cohorts, others have not detected higher frequencies of these autoantibodies in cancer patients compared to non-cancer controls (5). It remains to be determined if autoantibodies to DFS70/LEDGFp75 are more prevalent in PCa patients than in patients with other cancer types. Toward this goal, it would be important in future studies to determine the frequency of these autoantibodies in large cohorts of patients with different cancer types, individuals at high risk of developing cancer, and matched controls, using a combination of detection methods.

Our early observation that DFS70/LEDGFp75 is targeted by autoantibodies in some PCa patients is consistent with our more recent studies demonstrating its overexpression, both at the transcript and protein levels, in PCa tissues (8). This overexpression has been shown to be statistically significant, typically above a twofold difference compared to non-malignant tissues, and is not restricted to PCa since it has been observed in other cancer types (8, 9). Overexpression of DFS70/LEDGFp75 in malignant tissues could be induced by increased inflammation and oxidative stress in the microenvironment of the affected tissue, or by oncogenic viruses such as the human papilloma virus (HPV) (8, 9). A plethora of studies during the past decade by several laboratories has provided compelling evidence that when overexpressed in cancer cells, this autoantigen contributes to the upregulation and activation of a stress protective pathway that promotes tumor aggressive properties such as increased cell proliferation and survival, clonogenicity, migration, angiogenesis, tumor volume, and selective resistance to chemotherapy-induced cell death (8–10, 14). Thus, DFS70/LEDGFp75 is now considered an emerging tumor-associated antigen and stress oncoprotein that is relevant to multiple cancer types.

## DFS70/LEDGFp75 and Eye Diseases

Several studies have reported an association of anti-DFS70/LEDGFp75 autoantibodies with diverse inflammatory diseases of the eye including cataracts associated with atopic dermatitis, Vogt–Koyanagi–Harada (VKH) disease, sympathetic ophthalmia, Behcet’s disease, and atypical retinal degeneration [reviewed in Ref. (2, 10, 13)]. Notably, the original cDNA encoding the LEDGFp75 protein was identified from the screening of a lens epithelium-derived cDNA library using a serum from a patient with age-related cataract (13).

Anti-DFS70/LEDGFp75 antibodies (both human and rabbit) appear to have cytotoxic properties in LECs grown *in vitro*, suggesting a pathogenic role for the autoantibodies in the context of eye disease [reviewed in Ref. (13)]. While the mechanism of cytotoxicity was not clear, it was proposed that the antibodies bind to DFS70/LEDGFp75 released into the extracellular environment, preventing its re-entry into LECs where it acts as a pro-survival factor. It remains to be established, however, if these cytotoxic properties are specific for LECs since they have not been documented in other cellular or tissue models.

DFS70/LEDGFp75 has been shown to protect eye cells from various environmental stressors through the transcriptional activation of stress genes (13). It would be important, however, to determine in future studies if the humoral response to DFS70/LEDGFp75 in eye diseases or other inflammatory pathologies reflects an aberrant expression or function of this protein in the diseased tissue as a consequence of exposure to environmental stressors.

## What are the Autoantibodies to DFS70/LEDGFp75 Trying to Tell Us?

This question, first posed by our group a decade ago (2), is still elusive. In spite of the wealth of information on the biology and clinical associations of the DFS70/LEDGFp75 autoantibody–autoantigen system that has accumulated during the past 15 years, we still lack a clear understanding of this system. We suggest, however, that in our quest to elucidate the biological and clinical significance of humoral autoimmunity to anti-DFS70/LEDGFp75, we should take into account the following key observations:

(1) The nuclear DFS-IIF pattern produced by anti-DFS70/LEDG Fp75 autoantibodies is the most common ANA pattern in sera from patients with non-SARD diagnosis referred to clinical diagnostic laboratories (4, 5).
(2) Anti-DFS70/LEDGFp75 autoantibodies can be found in patients with diverse inflammatory conditions such as atopic dermatitis, alopecia areata, interstitial cystitis, asthma, pediatric chronic fatigue syndrome, miscellaneous non-SARD autoimmune and inflammatory diseases, eye diseases, and prostate cancer (1–7, 10). With a few exceptions, their frequency in these non-SARD inflammatory conditions usually ranges from 6 to 20%.
(3) These autoantibodies are typically IgG and can circulate at high titers, often reaching 1:5,120, in some “apparently healthy” individuals who stay healthy after years of follow up (6). This could suggest that they are natural antibodies.
(4) The C-terminal autoepitope targeted by anti-DFS70/LEDG Fp75 autoantibodies is a highly conserved, conformational, and functional region that serves as a hub for interactions with several chromatin-associated proteins (10–12).
(5) DFS70/LEDGFp75 is upregulated in a variety of cells and tissues in response to environmental stressors (e.g., UVB, hyperthermia, starvation, viral infections, cytotoxic drugs, etc.) and promotes cell survival by engaging in multiple complexes in the chromatin and transcriptionally activating stress protective, antioxidant, and inflammation-related genes (8–10, 13, 14).
(6) DFS70/LEDGFp75 is cleaved by caspases during apoptosis into fragments that contain the C-terminal autoepitope (2, 10).

We speculate that aberrant tissue manifestations of this autoantigen (i.e., overexpression, extracellular release, cell death cleavage, induction by viruses) under stressful and pro-inflammatory conditions could alter its immunogenicity, leading to the elicitation of the autoantibodies in susceptible individuals. For instance, the dynamic functions of the autoepitope domain of DFS70/LEDGFp75, which include interactions with multiple self- and non-self proteins to form shifting chromatin-associated complexes depending on cellular/molecular context, may influence its immunogenicity under inflammatory conditions, leading to autoimmune targeting. We reported previously that cleaved forms of this protein generated during apoptosis and persisting through secondary necrosis have a disrupted C-terminus that retains the autoepitope [reviewed in Ref. (2, 10)]. These aberrant forms of DFS70/LEDGFp75 enhance stress-induced cell death and may also exhibit increased immunogenicity in the context of tissue damage and inflammatory cell death. The extracellular release of DFS70/LEDGFp75, observed in LECs (13), could also make this protein the target of a humoral immune response if this release occurs in a pro-inflammatory microenvironment.

Depending on the context in which they arise, anti-DFS70/LEDGFp75 autoantibodies could play various roles. They may serve a protective role by removing DFS70/LEDGFp75 and its cleavage fragments from cellular debris generated during non-inflammatory apoptotic cell death. They could also play a pathogenic role by binding to extracellularly released DFS70/LEDGFp75 and preventing it from entering cells where it could activate a stress survival pathway. However, it would be fascinating to determine if autoantibody-mediated removal of DFS70/LEDGFp75 from extracellular circulation in the context of cancer or HIV infection could confer a protective advantage to patients due to the blockade of its pro-tumorigenic or HIV-IN binding functions, respectively, by the antibodies. These autoantibodies might also act as “sensors” or “reporters” of underlying, non-SARD inflammatory conditions associated with increased oxidative stress and aberrant expression or functions of DFS70/LEDGFp75 that alter its immunogenicity (10). Their presence in susceptible, “apparently healthy” individuals could be indicative of an undetected chronic inflammatory response.

It is plausible that these antibodies could be triggered by unidentified environmental factors. The recent observation that HPV activates DFS70/LEDGFp75 (9) raises the intriguing possibility that HPV vaccination or infection may be associated with overexpression of this autoantigen in a pro-inflammatory context. This could not only influence its cellular functions but also its immunogenicity.

## Conclusion

A better understanding of the context in which anti-DFS70/LEDGFp75 autoantibodies arise will require not only increased knowledge on DFS70/LEDGFp75 biology but also comprehensive information on individuals that test positive for these autoantibodies. This should include age, gender, health history, vaccination record, lifestyle, ethnicity, geographic location, and exposure to environmental stressors or xenobiotics. This might help determine if the presence of these autoantibodies could be linked to specific environmental factors that may influence the levels of oxidative stress in a particular tissue microenvironment, leading to tissue damage, altered DFS70/LEDGFp75 expression or function, and enhanced immunogenicity of this protein under pro-inflammatory conditions.

## Conflict of Interest Statement

The authors declare that the research was conducted in the absence of any commercial or financial relationships that could be construed as a potential conflict of interest.
